# How different effectors and action effects modulate the formation of separate motor memories

**DOI:** 10.1038/s41598-019-53543-1

**Published:** 2019-11-19

**Authors:** Raphael Schween, Lisa Langsdorf, Jordan A. Taylor, Mathias Hegele

**Affiliations:** 1 Justus Liebig University, Department of Psychology and Sport Science, Neuromotor Behavior Laboratory, Section Experimental Sensomotorics, Giessen, Germany; 2 Center for Mind, Brain and Behavior (CMBB) Universities of Marburg and Giessen, Giessen, Germany; 3 Princeton University, Department of Psychology Intelligent Performance and Adaptation Laboratory, Princeton, USA

**Keywords:** Cognitive neuroscience, Learning and memory, Motor control, Sensorimotor processing, Human behaviour

## Abstract

Humans can operate a variety of modern tools, which are often associated with different visuomotor transformations. Studies investigating this ability have shown that separate motor memories can be acquired implicitly when different sensorimotor transformations are associated with distinct (intended) postures or explicitly when abstract contextual cues are leveraged by aiming strategies. It still remains unclear how different transformations are remembered implicitly when postures are similar. We investigated whether features of planning to manipulate a visual tool, such as its visual identity or the environmental effect intended by its use (i.e. action effect) would enable implicit learning of opposing visuomotor rotations. Results show that neither contextual cue led to distinct implicit motor memories, but that cues only affected implicit adaptation indirectly through generalization around explicit strategies. In contrast, a control experiment where participants practiced opposing transformations with different hands did result in contextualized aftereffects differing between hands across generalization targets. It appears that different (intended) body states are necessary for separate aftereffects to emerge, suggesting that the role of sensory prediction error-based adaptation may be limited to the recalibration of a body model, whereas establishing separate tool models may proceed along a different route.

## Introduction

A hallmark of human motor skill is that we can manipulate a variety of different objects and tools. The apparent ease with which we switch between skilled manipulation of different tools requires that our motor system maintains representations of different sensorimotor transformations associated with them and retrieves these based on context^1,2^. For this to work, the brain is assumed to rely on contextual cues, i.e. sensations that allow the identification of the current context in a predictive manner (though see Lonini and colleagues^3^). This capacity has been investigated in dual adaptation experiments, where different cues are linked with different – often conflicting - sensorimotor transformations to determine the extent with which the cues enable the formation of separate visuomotor memories^4–21^. Whereas earlier studies have predominantly found that the posture or initial state of the body act as sufficient cues^11,14,19,22^, a number of more recent studies observed that distinct movement plans effectively separate memories for sensorimotor transformations^16,18,23–26^, even when these plans are not ultimately executed^16^.

As suggested by Sheahan and colleagues^16^, these findings can be unified under a dynamical systems perspective of neural activity^27^. Under this framework, neural states during movement execution are largely determined by a preparatory state prior to movement onset. Assuming that distinct states of the body as well as intended movements set distinct preparatory states, errors experienced during execution could therefore be associated with distinct neural states, thus establishing separate memories for novel transformations. While this idea is supported by recent findings showing that artificially induced neural states can serve as effective cues for the separation of newly formed sensorimotor memories^28^, these cues still pertain to intended states of the body such as visually observable movement outcomes or final postures.

In contrast, findings regarding more abstract context cues that are not directly related to the state of the body have been less consistent. For example, some studies have found color cues to enable dual adaptation^7^, while others have not^14,22^. Such inconsistent findings could be reconciled by the notion that motor memory can be subdivided into explicit and implicit types. These types are associated with different properties^29–33^ as well as neural processes^34,35^ and it is conceivable that they are also sensitive to different sets of context cues^9,26^. Thus, the effectiveness of color and other more abstract cues may be limited to cases in which participants become aware of the cues’ predictive power and develop distinct explicit action plans in response to them^8,9,26^.

In the use of modern electronic tools such as video game controllers or remotely controlled vehicles, however, there are many instances where similar postures or bodily states in general are associated with different sensorimotor transformations, yet skilled performers operate such tools effortlessly, suggesting that they have separate implicit memories of the respective transformations. Two features extrinsic to the body state that typically distinguish between different tools are (a) the visual representation of the effector that is controlled (as in the case of operating a drone or steering a virtual car by the same remote control) and (b) the action effect that can be produced by using the tool and thus that one strives to achieve. Thus, distinct preparatory states most likely incorporate features beyond bodily states, such as the identity of the tool which is operated and/or the nature of the intended action effect, suggesting that these may serve as cues to separate implicit memories.

Here, we considered the identity of the tool being controlled and the intended action effect as parts of a movement’s plan (i.e. its preparatory activity) and tested by which processes, implicit and/or explicit learning, these cues would allow for the development of separate motor memories. Based on a previous study by Howard and colleagues^14^, which showed that the visual orientation of a controlled object was modestly successful, we expected the visually perceived identity of a tool to constitute a relevant contextual cue in establishing separate implicit motor memories. To test this, participants practiced two opposing cursor rotations associated with different cursor icons or “tools”.

Our second approach was inspired by ideomotor theory, according to which actions are represented by their perceivable effects (see Stock and Stock^36^ for a review of its history). More specifically, it was based on a strong version of ideomotor theory claiming that effect anticipations directly trigger actions^37^. According to the theory of event coding^38^, effect anticipations are not limited to spatial properties, but can refer to any remote or distal sensory consequences anticipated in response to an action. The direct-activation hypothesis has received empirical support from neurophysiological studies showing that the mere perception of stimuli that had been established as action effects during a preceding practice phase were able to elicit neural activity in motor areas^39–41^. If we allow effect anticipations to be part of a neural preparatory state under the above view, this suggests that distinct action effects that a learner intends to achieve should allow distinct sensorimotor transformations to be associated with them. If confirmed, this would extend the state space relevant for the separation of implicit motor memory from physical to psychological dimensions^42,43^ and thereby potentially explain separation of this memory for movements with similar body states. To test this, we investigated how participants adapted to two opposing visuomotor cursor rotations when these were associated with different action effects.

Finally, we conducted a control experiment where we tested if the use of separate hands and thus clearly distinguishable bodily states would cue distinct implicit motor memories of the opposing visuomotor transformations. Given that different effectors can be considered different states of the body and that intermanual transfer of adaptation is limited^30,44,45^, we hypothesized that this would lead to clearly separate implicit memories for the two transformations.

## Results

### Experiment 1: Visual tools

**Figure 1:**
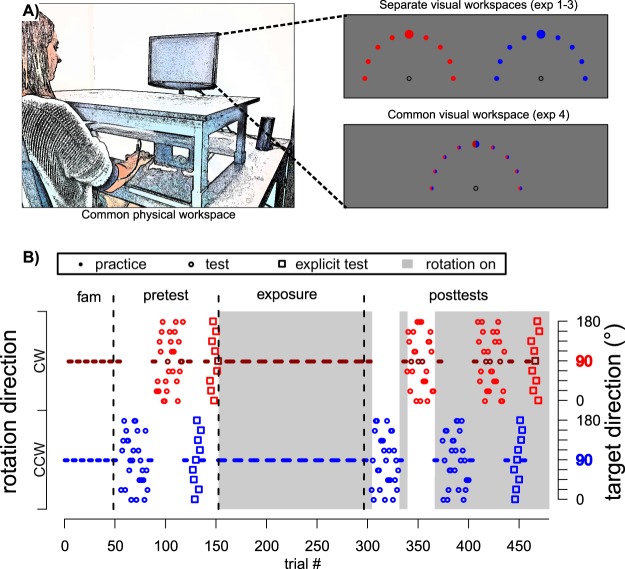
Experimental setup (**A**) and an exemplary task protocol (**B**). Adapted from Schween *et al*.^26^ under CC BY-4.0 license. Participants made rapid shooting movements, dragging a motion-captured sled attached to their index finger across a glass surface, to bring the different tools to a single target (indicated by larger size), which was oriented at 90° (with 0° corresponding to horizontal rightward movement and rotating counter-clockwise from 0°) and presented on a vertically-mounted computer screen. Continuous, online feedback was provided during practice, where cursor movement was veridical to hand movement in familiarization, but rotated 45° during rotation practice, with rotation sign and contextual cue level alternating jointly in blocks of 8 trials. Pre- and posttests without visual feedback tested generalization to different targets (practice target + generalization targets indicated by smaller size) with participants either instructed that the rotation was present (total learning) or absent (aftereffects) or judging the required movement direction using a visual support (explicit judgment), from which we inferred total, implicit and explicit learning, respectively.

**Figure 2:**
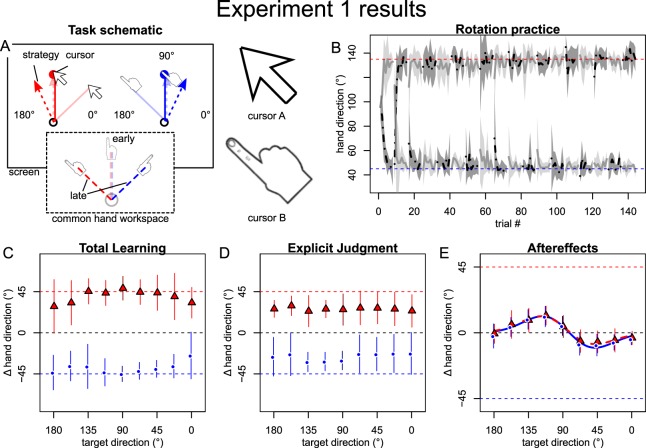
Results of experiment 1. (**A**) Opposing rotations were cued by different screen cursors and display in different parts of the visual workspace (VWS). (**B**) Light and dark grey lines and shades represent means and standard deviations of hand direction averaged over participants who began practice with CW rotation/left VWS or CCW rotation/right VWS, respectively (as this was counterbalanced). Participants quickly learned to compensate for both rotations, as indicated by grey lines approaching red and blue dashed horizontal lines marking perfect compensation. (**C**–**E**) Symbols with error bars represent across participant means and standard deviations of baseline-corrected posttest directions by generalization target (x-axis) and rotation sign/VWS (red vs. blue). Note that the pairing of cue level (i.e. cursor type) to rotation sign/VWS was counterbalanced across participants and the rotation-specific red and blue curves therefore contain both cue levels, but are distinct in VWS and rotation sign experienced. Thin, colored lines are individual participant values. Total learning (**C**) and explicit judgments (**D**) appeared to depend on the context cue, but implicit learning (**E**) only varied with test direction, and was fit better by a bimodal than a unimodal Gaussian (thick red and green lines), in line with local generalization around separate explicit movement plans.

The goal of experiment 1 was to determine if different visual tools could afford dual adaptation to conflicting sensorimotor transformations. In alternating blocks, participants practiced overcoming two opposing 45° visuomotor rotations by controlling two different visual tools, either a cartoon of a hand or an arrow cursor. (We note that these cues were also associated with separate regions of the visual workspace, i.e. left and right half of the screen in experiment 1 and 2 (Figs. 1A, 2A), but replication without this feature in experiment 4 suggests that it did not substantially affect results, which we therefore discuss as pertaining to the visual tool cue). The clockwise and counterclockwise perturbations were uniquely associated with either the hand or arrow cursor, counterbalanced across participants. To distinguish whether the context cue enabled separate memories to be formed and retrieved implicitly or through separate explicit motor plans^26^, we tested spatial generalization of learning under each cue differentially and dissociated total learning into explicit plans and implicit adaptation by a series of posttests^46^.

Within a few blocks of practice, all participants were able to compensate for the opposing rotations (Fig. 2B). Total learning, which is believed to comprise both explicit and implicit contributions, generalized broadly across directions in a pattern that is suggestive of dual adaptation (Fig. 2C). Much of the dual adaptation could be attributed to different explicit judgments (Fig. 2D) for each tool, while aftereffects appeared to contribute very little to the total learning (Fig. 2E). Note that, by “aftereffects”, we refer to changes in pointing performance in trials in which participants had been instructed that the rotations were absent.

What’s more, the pattern of generalization of the aftereffects appeared to be similar for each tool and exhibited a bimodal shape (Fig. 2E). We have previously shown that this bimodal aftereffect pattern can be explained by plan-based generalization^24–26^. Under this explanation, implicit adaptation accumulates locally, yielding Gaussian-like generalization peaks that center on the movement plan^24,25^. Dual adaptation paradigms produce opposing peaks that cancel out when the movement plans determining their centers overlap, but may persist locally when these generalization centers are separated by plans pointing in different directions^26^. The bimodal patterns in our present data therefore suggest that the cues did enable implicit dual adaptation indirectly by cuing separate explicit movement plans, similar to what we have already shown for the visual workspaces cue alone^26,47^. However, it appears there was no direct effect of the cues on implicit learning, since aftereffects across target directions were not contextualized, i.e. did not differ between the two cue levels.

As our primary goal was to determine if distinct tools could serve as cues to separate implicit visuomotor memories, we sought to further characterize the pattern of interference (or generalization) of the aftereffects. Here, we found that the mean aftereffects across generalization targets were fit better by the sum of two Gaussians (“bimodal” Gaussian) than a single Gaussian (ΔBIC: 16.1 for cued CW rotation, 17.0 for cued CCW rotation), which is consistent with our previous findings^26^. The gain parameters had opposite signs and their respective bootstrapped confidence intervals did not include zero (Table 1), suggesting that adaptive changes in response to both rotations were represented in each generalization function. The locations and signs of the peaks comply with what we previously explained by implicit adaptation for each of the two practiced rotations generalizing narrowly around the cue-dependent explicit movement plans^26^. Here, the bimodal curve can be thought of as the sum of these independent generalization functions, where the two modes reflect the two opposite peaks of the individual functions and interference is maximal at the practiced target. Importantly, confidence intervals of differences between bootstrapped parameters for the two curves included zero (Table 1), indicating that adaptation retrieved under the two context cue levels did not differ significantly. In summary, we take these results to show that visual tools did not cue separate implicit visuomotor memories, except indirectly, mediated by plan-based generalization around separate explicit movement plans.

**Table 1:** Parameters of generalization functions fit to across-participant mean aftereffects. Only parameters for the better-fitting model (unimodal or bimodal Gaussian) are shown.

Experiment	Cued rotation	Parameters				
		*„gain“ a1*	*„center“ b1*	*„gain“ a2*	*„center“ b2*	*„std.dev.“ c*
1: tool + VWS	CW	16.3° [10.5°; 85.1°]	105.5° [86.7°; 124.5°]	−13.2° [−83.3°; −5.6°]	66.0° [38.8°; 86.9°]	47.2° [31.7°; 56.0°]
	CCW	16.3° [10.4°; 98.1°]	103.5° [82.7°; 120.5°]	−15.4° [−97.4°; −8.2°]	62.5° [39.3°; 84.0°]	45.3° [33.7°; 52.4°]
	CW - CCW	[−79.2°; 66.7°]	[−28.4°, 35.4°]	[−65.8°; 81.9°]	[−37.5°; 38.2°]	[−13.6°; 13.7°]
2: effect + VWS	CW	10.2° [7.6°; 48.7°]	125.0° [94.9°; 133.4°]	−8.3° [−48.1°; −5.7°]	44.2° [35.0°; 82.2°]	40.4° [33.3°; 54.0°]
	CCW	13.4° [10.9°; 90.2°]	119.3° [91.6°; 128.3°]	−9.3° [−87.4°; −5.7°]	58.4° [36.5°; 91.8°]	42.3° [32.0°; 53.8°]
	CW - CCW	[−77.7°; 3.7°]	[−15.2°, 35.0°]	[−6.3°; 77.4°]	[−48.0°; 19.4°]	[−14.1°; 11.0°]
3: hands + VWS	CW	14.2° [11.4°; 17.5°]	108.6° [97.8°; 118.1°]			52.9° [44.2°; 61.8°]
	CCW	−16.5° [−20.6°; −13.1°]	75.2° [67.0°; 85.2°]			68.2° [52.8°; 86.2°]
4: tool	CW	13.3° [8.7°; 21.8°]	130.0° [119.1°; 137.0°	*a*_*2*_: −7.0° [−10.6°; −4.3°]	49.6° [34.0°; 58.1°]	29.5° [23.0°; 38.8°]
	CCW	10.0° [7.9°; 38.2°]	120.5° [92.5°; 125.4°]	−9.3° [−39.2°; 6.5°]	44.7° [36.2°; 76.3°]	36.0° [30.3°; 47.2°]
	CW – CCW	[−23.0°; 11.9°]	[−0.7°; 33.3°]	[−0.7°; 30.5°]	[−23.3°; 17.2°]	[−17.4°; 2.6°]
4: effect	CW	11.0° [7.0°; 14.9°]	126.6° [116.0°; 133.2°]	−4.6° [−9.5°; 1.2°]	30.3° [0.0°; 55.1°]	33.0° [−9.5°; 1.2°]
	CCW	9.6° [7.6°; 48.8°]	120.5° [93.5°; 126.8°]	−8.2° [−52.7°; 5.9°]	42.8° [28.8°; 72.8°]	34.2° [19.9°; 49.9°]
	CW - CCW	[−38.5°; 4.1°]	[−5.1°; 29.6°]	[−0.7°; 48.1°]	[−48.3°; 14.7°]	[−14.0°; 15.7°]

Square brackets contain 95% confidence intervals obtained by bootstrap resampling of participants and fitting to mean data. Differences between CW and CCW were calculated within each bootstrap sample to test for differences between aftereffects obtained under the cue levels. Abbreviations: Std. dev.: standard deviation. VWS: visual workspace.

### Experiment 2: Action effects

Motivated by ideomotor theory^37^, experiment 2 tested whether implicit memories of opposing transformations could be acquired and retrieved separately when they were associated with different intended effects of the motor action. For this purpose, participants were instructed that they should either “paint” or “explode” the target. The current effect was announced by an on-screen message at the beginning of each block and participants saw an animation of a brushstroke (paint-cue) or an explosion (explode-cue) where their cursor crossed the specified target amplitude and heard respective sounds. Again, we retained separate visual workspaces as in our previous experiments, but experiment 4 indicates that this additional cue of workspace separation did not substantially affect the observed results.

**Figure 3:**
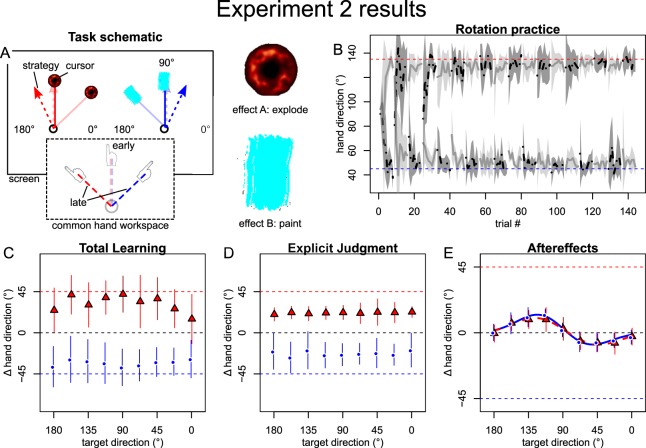
Results of experiment 2. (**A**) Opposing rotations were now cued by participants’ intention to either “paint” or “explode” the target. Target intentions were instructed by onscreen messages at the beginning of each block and supported by animations where the cursor crossed the target amplitude, accompanied by respective sounds. B: Similar to experiment 1, mean hand directions during practice (grey lines with shades indicating SDs) indicated that participants learned to compensate both rotations, quickly. (**C–E**) Baseline-corrected mean ( ± SD) and individual participant posttest directions for total learning (**C**) and explicit judgments (**D**) were specific to the contextual cue (red vs. blue) and generalized broadly across target directions (x-axis), while implicit aftereffects (**E**) remained cue-independent and only varied with target direction. It therefore appears that opposing transformations were learned specific to the intended effect only by explicit strategies and local, plan-based generalization around these (bimodal Gaussian fits indicated by thick red and blue lines in panel (**E**), but no distinct implicit memories were formed depending on the intended effect.

The results show no relevant qualitative differences compared to the visual tool cue used in experiment 1. Participants quickly compensated for both rotations during practice (Fig. 3B). Total learning and explicit judgments compensated for the rotations in a cue-dependent fashion and generalized broadly (Fig. 3C,D). Aftereffects (Fig. 3E) displayed a bimodal pattern (ΔBIC CW: 19.7, CCW: 18.8) that is visually similar to that of experiment 1. The oppositely signed peaks again complied with plan-based generalization and bootstrapped parameters indicated no difference between the curves for the different cue levels (Table 1). It therefore appears that separate action effects were ineffective in cuing separate implicit memories for the opposing transformations, except as mediated by spatially separate explicit movement plans.

### Experiment 3: Separate hands

As the first two context cues we tested were effective only via separate explicit strategies, we wanted to test a cue that would allow separate implicit memories to be created with relative certainty. Based on the findings that distinct body states cue separate implicit memories and that transfer of learning between hands is incomplete^30,44,48^, we reasoned that using different hands to practice the two rotations would be a promising approach. In experiment 3, a clockwise cursor rotation was therefore associated with left hand movements and visual display in the left half of the screen (left visual workspace), whereas a counterclockwise rotation was cued by right hand movements and right visual workspace display.

**Figure 4:**
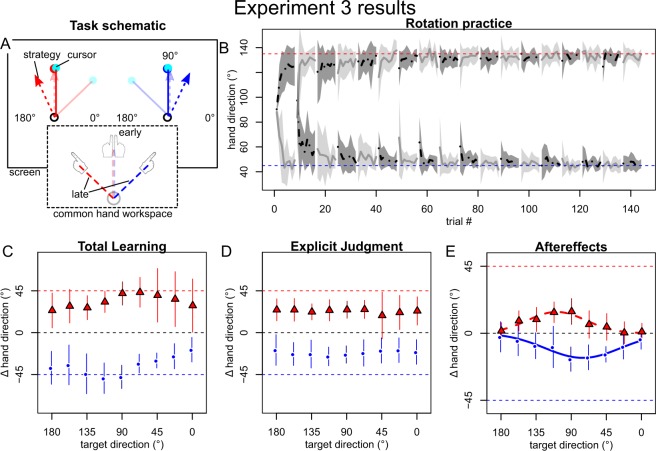
When using distinct hands to learn opposing transformations (**A**), participants also quickly compensated the rotation, as indicated by mean hand directions during practice (grey lines with SD shades) quickly approaching ideal compensation (red/green, dashed lines). (**C–E**) In contrast to experiment 1 and 2, across participant mean ( ± SD) directions now appeared specific to the cue level (red vs. blue symbols and thin lines) for aftereffects (**E**) in addition to total learning (**C**) and explicit judgments (**D**) and were best fit by a unimodal Gaussian (thick red and blue lines in **E**).

Similar to experiment 1 and 2, total learning and explicit judgments indicated that participants learned to compensate each rotation in a cue-specific fashion. Total learning at the practiced target almost completely compensated the rotation associated with each cue and relatively broad generalization to other targets occurred (Fig. 4C). Explicit judgments at the practiced target compensated about half of the cued rotation and also displayed a flat generalization pattern (Fig. 4D). Different than in the first two experiments, implicit aftereffects also showed a clear, cue dependent separation, with a single-peaked generalization pattern (Fig. 4E). This was supported by BIC being similar between single and bimodal Gaussian (ΔBIC CW: 1.9, CCW: 0.4, each in favor of the single Gaussian). The direction of the single Gaussians’ peaks depended on the hand cue, in line with each reflecting adaptation to the respective rotation practiced with that hand. Further, their locations were shifted off the practiced target in the direction consistent with plan-based generalization (Table 1). Interestingly, generalization appeared to be considerably wider than the peaks in the first two experiments and in previous studies^25,49^. Furthermore, we note that, despite separate implicit memories being established, implicit learning seemed incapable of accounting for the full cursor rotation, as it was supplemented by explicit strategies, in line with recent findings that the extent of implicit adaptation is limited^50^.

In summary, these results indicate that the cue combination of using separate hands in addition to the separate visual workspaces successfully cued separate visuomotor memories for both implicit adaptation and explicit strategies. As contextual separation of memories can be considered the inverse of transfer/interference between contexts, this is in line with findings suggesting that intermanual transfer of sensorimotor adaptation relies largely on strategies being flexibly applied across hand-context^30,44^ and that implicit learning transfers incompletely to the other hand^30,45,48^ (but see Kumar and colleagues^51^).

### Experiment 4

In experiments 1 to 3, we retained the visual workspace cue from our previous experiments^9,26^ in addition to the novel cue in focus. Furthermore, the cues were already present during baseline, where they did not have any predictive value. Drawing parallels to classical conditioning, one might suspect that the association of new implicit memories with the contextual cues in experiment 1 and 2 was prevented by either of two mechanisms: first, the presence of the visual workspace cue could have led the brain to ignore the predictive value of additional cues, similar to blocking^52^, or compound conditioning^53^. In this case one of our added cues could have actually enabled implicit dual adaptation, but be blocked by the visual workspace cue taking responsibility for the difference in context and directing adaptive changes towards the explicit route^26,47^. Second, similar to latent inhibition^54^, the lack of a predictive cue value during baseline could have prevented the implicit learning system from considering them even after their predictive value had been established. In Experiment 4, we control for those two possibilities by testing dual adaptation with only the visual tool or the action effects cue, presented exclusively during rotation practice and posttests. Because experiment 3 already found separate hands to effectively cue separate implicit memories, we did not include separate hands in this experiment.

Whereas, it appeared initially that there were indeed cue-dependent aftereffects in addition to the bimodal pattern, inspecting individual data revealed that the separation was driven by only a few participants (Supplementary figure S1). We think that the most likely explanation for this is that the respective participants ignored the instruction to abandon strategies in our aftereffects posttest due to misunderstanding and therefore excluded these participants for our main analyses (two out of 22 participants from the “tool” group and four out of 26 participants from the “effect” group, see methods). We discuss potential alternative interpretations in the Supplementary Text.

**Figure 5:**
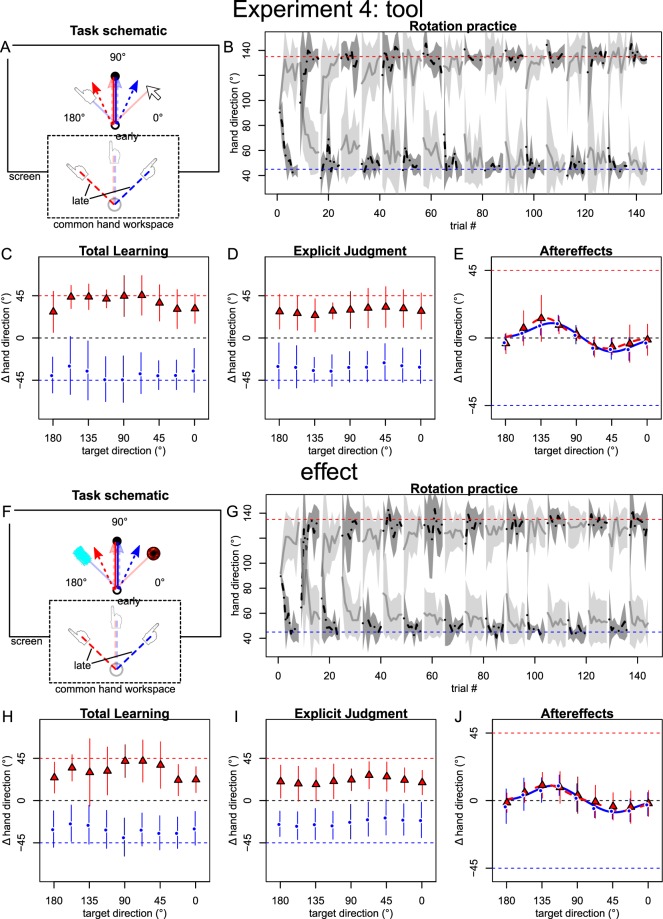
Results for the two cue-conditions in experiment 4, where cues were not shown during baseline and the additional workspace separation cue was absent. In panels E and J, means and fits were calculated excluding outliers (see methods & figures S1).

Without the participants thus labeled outliers, total learning in both cue conditions came close to full, cue-specific compensation of the rotations and generalized broadly across targets (Fig. 5C,H), while explicit judgments compensated about half of the rotations in a cue-specific fashion and also generalized broadly (Fig. 5D,I). Aftereffects again displayed opposing peaks on either side of the practiced target (Fig. 5E,J), although the fits under the action effect cue were a little more unstable and the confidence interval on the gain parameter for the negative peak now included zero (Table 1). Importantly, the fits still did not differ between the cue levels (Table 1).

In summary, experiment 4 did not provide convincing evidence that the tool and effect cue enabled separate implicit memories to be formed by the implicit adaptation mechanism that typically underlies cursor rotation aftereffects. If the modifications made in this experiment indeed induced separate implicit memories in a few participants, this was likely by a different mechanism (see Supplementary Text), which may be interesting to investigate in future experiments.

## Discussion

Based on the assumption that distinct preparatory states most likely incorporate features beyond bodily states, we considered the identity of a tool being controlled and the intended action effect as being part of a movement’s plan (i.e. its preparatory activity) and tested whether these cues would allow for the development of separate motor memories. We also distinguished between explicit and implicit forms of motor memories. Contrary to our expectation, neither distinct tools nor action effects appeared to produce separate implicit memories. Instead, the opposing transformations were represented in implicit memory only indirectly via local spatial generalization around explicit movement plans^16,23–26^. Consistent with previous findings^10,13^, it appears that separate implicit memories are inextricably linked to states of the movement or the body. Indeed, in a control experiment (experiment 3), we found that distinct aftereffects, an indicator for the development of separate implicit motor memories, formed when participants practiced the opposing visuomotor rotations with separate hands, which represent a strong cue within a bodily state space. Under the dynamical systems perspective invoked in the introduction^16,27^, these results would indicate that only past, current and future states of the body determine the preparatory state in areas relevant to implicit adaptation, while more abstract contextual cues that relate to the action but not to parts of the body, are processed differently.

Despite our findings, we know that people can manipulate a variety of complex tools with little cognitive effort, even if they share similar movement characteristics and workspaces. Thus, we would expect that humans are capable of separating and storing separate implicit memories based on cues that do not require distinct states of the body. This raises the question as to why studies have consistently failed to find contextual cues that do not depend on movement-related states^6,20,22^. One possibility lies in the way in which context was implemented during practice and testing: it is well possible that experimental parameters like the duration of practice, the frequency and schedule of change between transformation-cue-combinations^7,55^, or the way cues are presented in tests without visual feedback are responsible for the absence of implicit dual adaptation and it is a limitation of our study that we did not test different conditions. However, recent findings have shown that implicit adaptation in cursor rotation experiments approaches a fixed asymptote^50^, which suggests that even longer practice would not enable this implicit adaptation process to account for large-scale change in visuomotor relations. These findings thus align with ours in suggesting that learning transformations associated with tools and contexts may come about via mechanisms distinct from this canonical implicit adaptation.

What could be the nature of these mechanisms? Morehead and colleagues^56^ suggested that explicit movement plans are part of an action selection mechanism that can also operate implicitly, whereas implicit adaptation typically observed in cursor rotation experiments reflects a calibration mechanism for motor execution on a lower level. We speculate that the action selection level is where context-dependent learning of tool transformations occurs. Under this view, implicit, context-dependent learning could, for example, be achieved by proceduralization of strategies at the action selection level, in line with canonical views of skill learning^35,57^. Recent findings have shown that that new policies can be learned by exploration and reinforcement^58,59^, and that this is closely tied to explicit strategies^60,61^. A possibility is that explicit action selection tendencies may become proceduralized by associative learning. Consistent with this idea, recent findings indicate that stimulus-response associations in cursor rotation^62–64^ and visuomotor association paradigms^65^ can become “cached”, so that they are expressed by default in situations where cognitive processing resources are limited.

These roles we assign to action selection and execution are reminiscent of another canonical distinction in the motor learning literature, between the learning of body versus tool transformations^66–69^ giving rise to modifications of an internal representation of the body (body schema)^70,71^ and internal representations of tools, respectively^72,73^. Here, our results would suggest that aftereffects in standard cursor rotation experiments reflect a dedicated mechanism that keeps the system calibrated to minor changes in the transformations of the body, and is therefore sensitive to context regarding the state of the body, but not other aspects of the motor environment. Notably, limiting standard implicit adaptation to a body model does not necessarily contradict the idea that internal models and the cerebellum underlie tool transformations^1,12,74^. Recent neuroimaging and patient studies indicate that cerebellum-based internal models support not only the implicit^75^, but also the explicit component of visuomotor adaptation^76,77^. A possibility is that internal models support the selection of suitable actions by simulating their hypothetical outcomes^78^.

Within the theory of event coding (TEC)^38^ mentioned in the introduction, our findings can be explained along a similar route: TEC acknowledges that neural signatures underlying perception and action need to be distinct at the far ends of this continuum and common coding and effect-based representation of actions can therefore only occur on an intermediate level^38^. As such, our results would place implicit adaptation to cursor rotations towards the action side of processing, thus explaining why separate action effects did not enable separate learning in our study, but the use of separate effectors did.

Throughout this work, we chose to call visual tools, action effects and the limb used for execution a “contextual cue”, whereas we did not consider the different targets contextual cues that we utilized to test generalization. A more holistic view would be that the brain represents a multidimensional state space composed of psychological and physical dimensions and contextual separation is achieved by separation in any of these dimensions. Our choice to define a visuomotor memory as representing the physical space of the experiment is therefore an arbitrary one. Eventually, the question of how to define “contextual cue” therefore becomes the same as which contextual cues enable the formation of separate memories in which ways. In this sense, our findings mean that adaptation of the implicit body model reflected in aftereffects only responds to contextual cues that are directly related to the state of the body, whereas a supposed action selection component can in principle account for any contextual cue provided that the cue is either subject to overt attention or supported by a previously reinforced association. This may be taken to suggest that separate neural correlates of these learning processes represent our percept of the world and body in state spaces of different dimensionality.

One may suspect that our findings depend critically on the size of the visuomotor rotation investigated and that reducing the size of the rotation would have reduced explicit learning through error awareness, thereby boosting implicit learning. Whereas rotation size does modulate explicit learning^79^, it is currently unclear if explicit strategies modulate implicit learning, with some findings suggesting an influence^80^, whereas others suggest implicit learning to be relatively robust against strategy and rotation magnitude^79,81^. Nevertheless, it remains a possibility that smaller rotations could reveal context effects on implicit learning – an avenue for future research.

In conclusion, our results show that neither the visual identity of two digital tools nor the effect intended by their use did allow the formation of separate implicit motor memories under the conditions studied. While extended practice under different conditions may lead to more implicit learning, we take our results to suggests that this may not come about by one implicit internal model becoming differentiated into two, but rather by learners acquiring new action selection tendencies. These tendencies may be explicit, initially, and become habituated with practice^62–65^, or they could be acquired incidentally depending on practice conditions^82^, explaining how we handle those tools with little cognitive effort.

## Methods

A total of one-hundred human volunteers participated in the study. All participants provided written, informed consent as approved by the local ethics committee of the Department of Psychology and Sport Science of Justus-Liebig-Universität Giessen and experiments were conducted in accordance with the relevant guidelines and regulations. To be included in analysis, participants had to be between 18 and 30 years old, right handed, have normal or corrected to normal visual acuity and were not supposed to have participated in a similar experiment, before. We therefore excluded 8 participants (5 for not being clearly right-handed according to the lateral preference inventory^83^, 2 for failing to follow instructions according to post-experimental standardized questioning, one for exceeding our age limit), giving us a total of 18 analyzed participants in experiment 1, 17 in experiment 2, and 20 in experiment 3.

### Apparatus

The general task and protocol were similar to those described in our previous study^26^. Participants sat at a desk, facing a screen running at 120 Hz (Samsung 2233RZ), mounted at head height, 1 m in front of them (Fig. 1A). They moved a plastic sled (50 × 30 mm base, 6 mm height) strapped to the index finger of their right hand (and left hand, respectively, in experiment 3), sliding over a glass surface on the table, with low friction. A second tabletop occluded vision of their hands. Sled position was tracked with a trakSTAR sensor (Model M800, Ascension technology, Burlington, VT, USA) mounted vertically above the fingertip, and visualized by a custom Matlab (2011, RRID:SCR_001622) script using the Psychophysics toolbox (RRID:SCR_002881)^84^, so that participants controlled a cursor (cyan filled circle, 5.6 mm diameter or specific cursors in *experiment 1*).

### Trial types

Trials began with arrows on the outline of the screen guiding participants to a starting position (red/green circle, 8 mm diameter) centrally on their midline, about 40 cm in front of their chest. Here, the cursor was only visible when participants were within 3 mm of the start location. After participants held the cursor in this location for 500 ms, a visual target (white, filled circle, 4.8 mm diameter) appeared at 80 mm distance and participants had to “shoot” the cursor through the target, without making deliberate online corrections. If movement time from start to target exceeded 300 ms, the trial was aborted with an error message.

Participants experienced 3 types of trials. On *movement practice* trials, they saw the cursor moving concurrently with their hand. Here, cursor feedback froze for 500 ms, as soon as target amplitude was reached. On *movement test* trials, we tested behavior without visual feedback meaning that the cursor disappeared on leaving the start circle. On *explicit judgment* trials^46^, we asked participants to judge the direction of hand movement required for the cursor to hit the target, without performing a movement. For this purpose, participants verbally instructed the experimenter to rotate a ray that originated in the start location to point in the direction of their judgment. During judgments, they were asked to keep their hand supinated on their thigh in order to discourage them from motor imagery. Accordingly, moving towards the start position was not required.

### General task protocol

The experiment consisted of four phases: familiarization, pretests, rotation practice and posttests (Fig. 1B). *Familiarization* consisted of 48 movement practice trials to a target at 90° with veridical cursor feedback, thus requiring a movement “straight ahead”. The cue condition (see *Specific experiments*) alternated every 4 trials, with condition order counterbalanced across participants. *Pretests* contained movement practice tests and explicit judgment tests to establish a baseline for subsequent analysis. We tested generalization to 9 target directions from 0° to 180° at the amplitude of the practice target. We obtained one set per cue level, which in turn consisted of 3 blocks of randomly permuted trials to each of the 9 target directions for movement tests and one such block for explicit judgment tests. The sets were interspersed by blocks of 8 practice movements (4 per cue level) to the 90° target, to refresh participants’ memory (Fig. 1B). There were thus 104 trials in the pretests: 2 × 27 movement tests, 2 × 9 explicit tests, 4 × 8 movement practice trials.

In the subsequent *rotation practice* phase, participants performed 144 trials toward the practice direction with cursor movement being rotated relative to hand movement by 45°. The sign of the rotation switched between clockwise (CW) and counterclockwise (CCW) depending on the context condition, which here alternated every 8 trials. Before we first introduced the cursor rotation, we instructed participants that they would still control the cursor, but that the relation between the direction of hand and cursor movement would be changed and that this changed relation would be signaled by a red, instead of the already experienced green start circle.

Rotation practice was followed by a series of posttests to dissociate implicit, total and explicit learning. The *posttests* were structured like the pretests, except that the movement tests were repeated twice: the first repetition tested for implicit aftereffects by instructing participants to assume the rotation was switched off, reasoning that this would induce them to abandon potential aiming strategies and aim directly at the target. The second repetition tested for total learning by instructing them that the rotation was switched on. For the explicit judgment tests, the rotation was instructed as switched on to test for explicit knowledge about the cursor rotation. Throughout the experiment, the presence or absence of the rotation was additionally cued by the color of the starting position (green = switched off, red = switched on), which participants were repeatedly reminded of.

### Specific experiments

The experiments differed in the type of contextual cue associated with the opposing cursor rotations. In all experiments, the rotation sign was associated with a visual workspace cue, meaning that start, cursor and target locations were presented with a constant y-axis shift of ¼ screen width (Fig. 1A). CW cursor rotation was always associated with display in the left half of the screen while CCW rotation was displayed in the right half. Hand movements were performed in a joint central workspace. We retained this for consistency with our previous experiments, where we found that it did not cue separate implicit memories and instead produced a pattern consistent with plan-based generalization^9,26^. Our main interest was thus on whether the added cues would enable separate implicit memories to be formed.

In *experiment 1*, the added cue was the visual identity of the cursor: participants either saw a hand icon or an arrow cursor. These cursor types were associated with the existing combination of visual workspace and cursor rotation in a way that was constant within, but counterbalanced across participants. As the cursor was visible once participants were in the vicinity of the start location, they could anticipate the upcoming rotation based on the cue in all movement trials. On explicit posttests, the cursor cue was attached to the far end of the ray that signaled participants response.

The added contextual cues for *experiment* 2 were two different action effects: Participants were instructed that they would have to either “explode” the target or “paint” it. The effect that participants should intend was prompted by a screen message at the beginning of each block (German: “Zerstören!” or “Anmalen!”). Accordingly, an animated explosion or brushstroke appeared at the location where the cursor crossed target amplitude, accompanied by respective sounds. As in experiment 1, action effects were fixed to visual workspaces and rotation direction within, but counterbalanced across participants. During movement tests without feedback, participants received the onscreen message before each block and the audio was played to remind them of the intended action effects.

In *experiment 3*, the additional cue was the hand used to conduct the movement, where we always associated the left hand with the left visual workspace and the right hand with the right visual workspace. At the beginning of each block, participants were prompted about which hand to use by an onscreen message and we asked them to rest the idle hand in their lap.

In *experiment 4*, participants were randomly assigned to two groups that received the “tool” or the “action effect” cue, respectively. Participants now saw all movements in a common visual workspace. Furthermore, context cues were not given during baseline and pretests and were only introduced together with the rotation. Matching of rotation sign to cue was counterbalanced across participants, as was the order of rotations presented to them. On aftereffect posttest, some participants displayed behavior that appeared to differ categorically from the majority of the group. We therefore labeled those participants whose mean aftereffects across all targets under either of the cues was outside two standard deviations of the group mean as outliers and excluded them from the main analyses. We show these participants’ performance and discuss alternative treatments in the Supplementary Material.

### Data analysis

We performed data analysis and visualization in Matlab (RRID:SCR_001622) and R (RRID:SCR_001905). X- and y-coordinates of the fingertip were tracked at 100 Hz and low-pass filtered using MATLAB’s “filtfilt” command (4-th order Butterworth, 10 Hz cutoff frequency). We then calculated the movement-terminal hand direction as the angular deviation of the vector between start and hand location at target amplitude and the vector between start and target. We excluded the following percentages of trials for producing no discernible movement endpoints (usually because the trial was aborted): experiment 1: 5.3%, experiment 2: 4.4%, experiment 3: 3.6%, and an additional total of 24 trials for producing hand angles more than 120° from the ideal hand direction on a given trial. Explicit direction judgments were calculated as the deviation between the vector connecting the start position with the target and the participants’ verbally instructed direction judgement. To obtain our measures of aftereffects, total learning, and explicit judgments, we calculated the median of the three repetitions per target in each pre- and posttest, under each cue level, for each participant, and subtracted the individual median of pretests from their respective posttests to account for any biases^85^. As main outcome measure, we therefore report direction changes from pretest to the different posttests types, depending on test target direction and context cues.

### Statistical analysis

As we were interested in whether or not the contextual cues enabled separate implicit memories, we focused on aftereffects and only report explicit judgments and total learning descriptively, for completeness. Furthermore, as generalization of explicit judgments appears to strongly depend on methodological details^26,49^, we would not claim universal validity of our findings in this respect. In our main analysis, we aimed to infer whether implicit aftereffects assessed under each cue reflected only the cued transformation, or both, and if aftereffects differed depending on the cue level. We therefore fit two candidate functions to the group mean aftereffect data obtained under each cue, respectively. In line with our previous reasoning^26^, we chose a single-peaked Gaussian to represent the hypothesis that aftereffects reflected only one learned transformation:

$$y=a\ast {e}^{-\frac{{(x-b)}^{2}}{{c}^{2}}}$$

Here, *y* is the aftereffect at test direction *x*. Out of the free parameters, *a* is the gain, *b* the mean and *c* the standard deviation.

The hypothesis that aftereffects reflected two transformations was represented by the sum of two Gaussians:

$$y={a}_{1}\ast {e}^{-\frac{{(x-{b}_{1})}^{2}}{{c}^{2}}}+{a}_{2}\ast {e}^{-\frac{{(x-{b}_{2})}^{2}}{{c}^{2}}}$$

For this, we assumed separate gains *a*_1_; *a*_2_ and means *b*_1_; *b*_2_ but a joint standard deviation *c*. For fitting, we used Matlab’s “fmincon” to maximize the joint likelihood assuming independent Gaussian likelihood functions for the residuals. We restarted the fit 100 times from different values selected uniformly from the following constraints: −180° to 180° on *a*, 0° to 180° on *b*-parameters, 0° to 180° on *c*, and subsequently compared the fits with the highest likelihood for each model by Bayesian information criterion (BIC), calculated as:

$$BIC=\,\mathrm{ln}(n)\ast k+2\ast \mathrm{ln}(lik)$$

with *n* being the number of data points, *k* number of free parameters and *lik* the joint likelihood of the data under the best fit parameters.

In order to test if the generalization functions thus obtained differed significantly between the two context cues used in each experiment, respectively, we created 10000 bootstrap samples by selecting participants randomly, with replacement, and fitting on the across subject mean, starting from the best fit parameters of the original sample. For each sample, we calculated the difference between parameters obtained for each cue level. We considered parameters to differ significantly if the two-sided 95% confidence interval of these differences, calculated as the 2.5^th^ to 97.5^th^ percentile, did not include zero.

## Supplementary information

Experiment 4 - individual participants

## Data Availability

The datasets generated and analyzed during the current study are available from the corresponding author on reasonable request.
