# Supplementary material for: How different effectors and action effects modulate the formation of separate motor memories
Source: Sci Rep. 2019 Nov 19;9:17040. doi: 10.1038/s41598-019-53543-1 (PMC6864246; doi:10.1038/s41598-019-53543-1)
Supplement: Supplementary file 1 — Experiment 4 - individual participants [file 41598_2019_53543_MOESM1_ESM.pdf]

How different effectors and action effects modulate the formation of separate motor memories

Raphael Schween, Lisa Langsdorf, Jordan A Taylor, Mathias Hegele

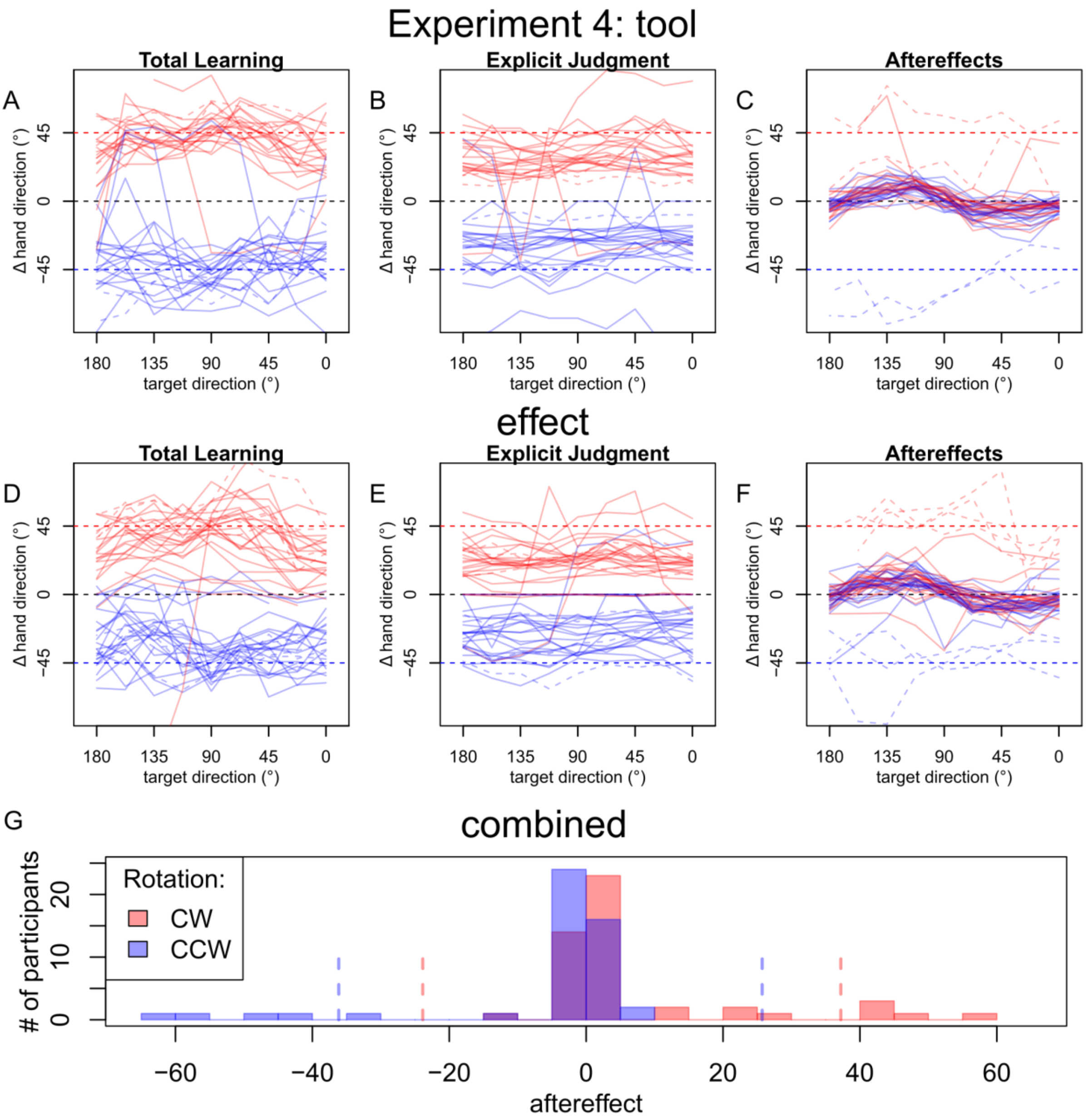

Figure S1: Individual participants' data for the two groups of experiment 4 ("tool" group: panel A-C; "effect" group, panel D-F). Data from outlier participants are shown as dashed instead of solid lines. Panel G illustrates our criterion for labeling outliers: Histogram of individual aftereffects, averaged across generalization directions. Dashed, vertical lines mark 2 standard deviations from group mean for the CW and CCW rotation cue, respectively. Participants whose performance under at least one of the cues lay outside respective boundaries were labeled outliers and excluded from the main analysis.

Supplementary figure S1 depicts individual participants' performances for experiment 4, with the participants labeled outliers marked by dashed instead of solid lines. A straightforward explanation for these participants' behavior is that they misunderstood or ignored the instruction that the cursor

rotation was removed in our posttest for aftereffects. The fact that such outliers only occurred in a discernible quantity in experiment 4 may be rooted in minor methodological changes from the collection of experiment 1 and 2 to experiment 4 (e.g. different experimenters).

However, we cannot exclude the possibility that these participants indeed learned the rotations differently. Assuming the aftereffects of the outliers reflect truly implicit learning, we note that aftereffect magnitude in these participants approaches full compensation of the 45° rotations and their generalization function does not appear to substantially fall off, even at 90° from the practiced target. This is in marked contrast to the known properties of implicit adaptation, which asymptotes around 20°[1,2] and generalizes narrowly without offset[3,4], even in single rotation practice[5,6]. Furthermore, behavior appears to differ categorically between participants, or even within participants between cue conditions. We therefore reason that if aftereffect in outliers are not due to a misinterpretation of task instructions, they likely represent learning by a separate mechanism that is different from canonical, sensory prediction error-driven aftereffects. A candidate mechanism would be the implicit acquisition of action selection tendencies. Recent experiments have highlighted that explicit strategies can become implicit with practice[7–10]. As this habituation likely relies on associative learning[11,12] it is conceivable that the changes inspired by classical conditioning, that we made in experiment 4, led to such implicit action selection strategies acquired either by habituation or incidental learning[13] in some participants.

Importantly, whether the participants with large aftereffects misunderstood the posttest or learned by a different mechanism, this does not change our main conclusion that the tool or action effect cue did not enable dual adaptation by canonical, sensory prediction error-driven aftereffects.

Supplementary References

1. Kim, H. E., Morehead, J. R., Parvin, D. E., Moazzezi, R. & Ivry, R. B. Invariant errors reveal limitations in motor correction rather than constraints on error sensitivity. *Commun. Biol.* **1**, 19 (2018).

2. Morehead, J. R., Taylor, J. A., Parvin, D. E. & Ivry, R. B. Characteristics of Implicit Sensorimotor Adaptation Revealed by Task-irrelevant Clamped Feedback. *J. Cogn. Neurosci.* **29**, 1061–1074 (2017).

3. McDougle, S. D., Bond, K. M. & Taylor, J. A. Implications of plan-based generalization in sensorimotor adaptation. *J. Neurophysiol.* **118**, 383–393 (2017).

4. Day, K. A., Roemmich, R. T., Taylor, J. A. & Bastian, A. J. Visuomotor learning generalizes around the intended movement. *eNeuro* **3**, e0005-16 (2016).

5. Heuer, H. & Hegele, M. Generalization of implicit and explicit adjustments to visuomotor rotations across the workspace in younger and older adults. *J. Neurophysiol.* **106**, 2078–85 (2011).

6. Poh, E. & Taylor, J. A. Generalization via superposition: Combined effects of mixed reference frame representations for explicit and implicit learning in a visuomotor adaptation task. *J. Neurophysiol.* 1953–1966 (2019). doi:10.1152/jn.00624.2018

7. McDougle, S. D. & Taylor, J. A. Dissociable cognitive strategies for sensorimotor learning. *Nat. Commun.* **10**, (2019).

8. Hardwick, R. M., Forrence, A. D., Krakauer, J. W. & Haith, A. M. Time-dependent competition between habitual and goal-directed response preparation. *bioRxiv* 201095 (2018). doi:10.1101/201095

9. Huberdeau, D. M., Krakauer, J. W. & Haith, A. M. Practice induces a qualitative change in the memory representation for visuomotor learning. *bioRxiv* (2017). doi:10.1101/226415

10. Leow, L.-A., Marinovic, W., Rugy, A. De & Carroll, T. J. Task errors drive memories that improve sensorimotor adaptation. *bioRxiv* 1–29 (2019).

11. Holland, P. J., Codol, O. & Galea, J. M. Contribution of explicit processes to reinforcement-based motor learning. *J. Neurophysiol.* **119**, 2241–2255 (2018).

12. Codol, O., Holland, P. J. & Galea, J. M. The relationship between reinforcement and explicit strategies during visuomotor adaptation. *Sci. Rep.* **8**, 9121 (2018).

13. Lee, T. G., Acuña, D. E., Kording, K. P. & Grafton, S. T. Limiting motor skill knowledge via incidental training protects against choking under pressure. *Psychon. Bull. Rev.* 1–12 (2018). doi:10.3758/s13423-018-1486-x
